# TRPP2 and TRPV4 Form an EGF-Activated Calcium Permeable Channel at the Apical Membrane of Renal Collecting Duct Cells

**DOI:** 10.1371/journal.pone.0073424

**Published:** 2013-08-16

**Authors:** Zhi-Ren Zhang, Wen-Feng Chu, Binlin Song, Monika Gooz, Jia-Ning Zhang, Chang-Jiang Yu, Shuai Jiang, Aleksander Baldys, Pal Gooz, Stacy Steele, Grzegorz Owsianik, Bernd Nilius, Peter Komlosi, P. Darwin Bell

**Affiliations:** 1 Ralph H. Johnson Veterans Affairs Medical Center, Charleston, South Carolina, United States of America; 2 Department of Medicine, Medical University of South Carolina, Charleston, South Carolina, United States of America; 3 Departments of Pharmacy and Cardiology of the 2^nd^ Affiliated Hospital, Department of Pharmacology, Key Laboratories of Education Ministry for Myocardial Ischemia and Treatment, Harbin Medical University, Harbin, P. R. China; 4 Department of Cellular and Molecular Medicine, Laboratory of Ion Channel Research, Campus Gasthuisberg, KU Leuven, Leuven, Belgium; University of Houston, United States of America

## Abstract

**Objective:**

Regulation of apical calcium entry is important for the function of principal cells of the collecting duct. However, the molecular identity and the regulators of the transporter/channel, which is responsible for apical calcium entry and what factors regulate the calcium conduction remain unclear.

**Methods and Results:**

We report that endogenous TRPP2 and TRPV4 assemble to form a 23-pS divalent cation-permeable non-selective ion channel at the apical membrane of renal principal cells of the collecting duct. TRPP2\TRPV4 channel complex was identified by patch-clamp, immunofluorescence and co-immunprecipitation studies in both principal cells that either possess normal cilia (cilia (+)) or in which cilia are absent (cilia (-)). This channel has distinct biophysical and pharmacological and regulatory profiles compared to either TRPP2 or TRPV4 channels. The rate of occurrence detected by patch clamp was higher in cilia (-) compared to cilia (+) cells. In addition, shRNA knockdown of TRPP2 increased the prevalence of TRPV4 channel activity while knockdown of TRPV4 resulted in TRPP2 activity and knockdown of both proteins vastly decreased the 23-pS channel activity. Epidermal growth factor (EGF) stimulated TRPP2\TRPV4 channel through the EGF receptor (EGFR) tyrosine kinase-dependent signaling. With loss of cilia, apical EGF treatment resulted in 64-fold increase in channel activity in cilia (-) but not cilia (+) cells. In addition EGF increased cell proliferation in cilia (-) cell that was dependent upon TRPP2\TRPV4 channel mediated increase in intracellular calcium.

**Conclusion:**

We conclude that in the absence of cilia, an EGF activated TRPP2\TRPV4 channel may play an important role in increased cell proliferation and cystogenesis.

## Introduction

One feature of the transient receptor potential (TRP) protein family is the propensity to form multimeric and heteromeric channel complexes. It has been reported that TRPP1 and TRPP2 associate to form a functional complex in cilia and that this complex functions to sense ciliary bending and to induce Ca^2+^ influx through TRPP2. TRPP1 is a large protein that has been proposed to have mechanosensory functions while TRPP2 is a calcium (Ca^2+^) permeable non-selective cation channel [1]. Both TRPP1 and TRPP2 are expressed at the apical membrane, in cilia, as well as other locations in epithelial cells. Mutations in TRPP1 and TRPP2 cause autosomal dominant polycystic kidney disease (ADPKD) [2]. Several studies, using heterologous expression systems, demonstrated that TRPP2 interacts with TRPC1 to form a channel complex [3–5]. This channel complex functions as a G-protein-coupled receptor (GPCR)-activated channel with the distinct biophysical properties from either TRPP2 or TRPPC1 [3]. Using constructs and expression systems, TRPP2 has been shown to also interact with TRPV4 to form a channel complex that has thermosensory properties [6]. Using atomic force microscopy, Stewart and co-workers demonstrated that TRPP2 and TRPV4 form a heterotetramer with stoichiometry of 2:2 which is the same stoichiometry reported for the TRPP2\TRPPC1 channel complex [7]. Importantly, it is currently not known whether endogenous TRPP2 and TRPV4 assemble to form a function channel complex, what regulates this channel complex, and what role(s) this putative TRPP2\TRPV4 channel complex may play in the physiological and pathophysiological processes.

A common feature of autosomal recessive polycystic kidney disease (ARPKD) in humans and mice is a distension of the renal collecting tubules caused by a localized proliferation and aberrant secretion of growth factors by epithelial cells [8]. Interestingly, cystic fluid has been shown to contain biologically active ligands for the EGFR, such as EGF and TGF-α [9]. It is well established that the EGF receptor (EGFR), which is normally located to the basolateral membrane, is mislocalized to the apical membrane of renal epithelial cells in PKD. Wilson and coworkers have reported that the renal epithelial cell apical receptor in ADPKD is a heterodimerization of EGFR (HER-1) with HER-2 (neu/ErbB2) [10]. The role of apical EGFR in the initiation and progression of renal cystic development remains unclear.

At the present time there is little information concerning the characteristics of the native apical Ca^2+^ channel in principal cells of the collecting duct. Clearly TRPP2 is present and functions as a Ca^2+^ channel at the apical membrane, however, there is no information currently available on whether endogenous TRPP2 forms multimeric complexes with other endogenous TRP channels (besides TRPP1) in principal cells of the collecting duct. In addition, there is a lack of information regarding what controls or regulates this channel complex. Previous work, again in heterologous expression systems, has found that EGFR activation enhances the activity of TRPP2 [11]. Whether EFGR influences Ca^2+^ entry in native cells is also unknown. Therefore, the purpose of this study was to perform a biophysical characterization of the apical cation (Ca^2+^) channel in principal cells of the collecting duct, to determine the molecular identity of this channel and to assess the regulation of this channel by the epidermal growth factor receptor, as well as to determine the functional role of this channel in a model of PKD.

## Materials and Methods

### Cell lines and reagents

The *orpk* cilia (+) and cilia (-) collecting duct cell lines were generated as described previously [12]. These cells were derived from a hypomorph to the *ift88/Tg737* gene that encodes for the intraflagellar transport protein polaris. Therefore the loss of cilia is almost, but not entirely complete but for convenience these cells are referred to as cilia (-). The wild-type *Tg737* gene was reintroduced into this cell line, which corrected the ciliary defect, creating the *orpk* cilia (+) immortalized collecting duct cell line. Cells were cultured on permeable support attached to *Snapwell* (Corning Costar Co.) under the conditions similar to those described previously [13]. To facilitate cell polarization and to promote differentiation and SV40 large-T antigen inactivation cells were incubated under nonpermissive conditions at 39°C in the absence of interferon-γ for 10-14 days. Unless otherwise noted, all reagents were purchased from Sigma Aldrich (USA).

### Electrophysiology

Prior to experiments, cells were thoroughly washed with the Ringer’s solution containing (in mM) 140 NaCl, 5 KCl, 1.5 CaCl_2_, 1 MgCl_2_, and 10 HEPES (pH 7.4 with NaOH). Cell-attached and inside-out patch-clamp were performed at room temperature (22-24°C). Pipettes had resistances in a range of 5-10 MΩ when filled with Ringer’s solution. High-resistance seals (16-32 GΩ) were obtained for all recordings and the currents were recorded with an Axopatch 200B amplifier (Molecular Devices, Sunnyvale, CA, USA), and data were filtered at 1 kHz and sampled at 5 KHz. Prior to analysis, the single-channel traces were further filtered to 100 Hz and the baseline-shifts in some records were corrected manually using pClamp 10 (Molecular Devices, Sunnyvale, CA, USA). The single-channel amplitude was constructed by all-point amplitude histogram and the histograms were fit using multiple Gaussians and optimized using a simplex algorithm. *P*_*O*_ was calculated as *P*_*O*_ = *NP*_*O*_/*N*, where N (N was estimated by the current amplitude histogram during at least 5 min recording period) is the apparent number of active channels in the patch. The i-V relationships were constructed using the single-channel amplitude (i) at the indicated voltages (V_M_), as a function of voltages and the slope conductance was fit by linear regression by SigmaPlot software (Jandel Scientific, CA, USA). For ion selectivity experiments, an agar bridge (3 M KCl in 3% agar) served as ground and data are corrected for junction potentials and the conductance of inward and outward currents was fit separately and the reversal potentials were determined by linear regression around zero-current value. The junction potential was 7.71 ± 0.62 mV (n = 7) while 140 mM NaCl of the Ringer’s solution was replaced by equimolar BaCl_2_. Replacement of Na^+^ by equimolar Ba^2+^ did not alter the osmotic pressure (310 mOsm/kg for Na^+^ vs. 312 mOsm/kg for Ba^2+^). Reversal potentials for Na^+^ and for each test cation were used to calculate relative permeability [cation x- to-Na^+^ permeability (P_x_/P_Na_^+^)] according to the modified Goldman-Hodgkin-Katz equation:

$$\frac{P_{x}}{P_{\text{Na}}}=\frac{{\left[{\text{Na}}^{+}\right]}_{\text{r}}10^{zF\Delta V_{r}/RT}-{\left[{\text{Na}}^{+}\right]}_{\text{t}}}{{\left[x^{+}\right]}_{\text{t}}}$$

Where [Na^+^]_r_ and [Na^+^]_t_ are the concentrations of Na^+^ in the reference and test solutions, respectively; [$x^{+}$]_t_ is the concentration of cation x in the test solution; ΔV_r_ is the change in reversal potential; z is the valence; R is the gas constant; T is the absolute temperature; and F is the Faraday constant [14].

### Measurement of apical membrane calcium permeability by Mn^2+^ quenching in Fura-2-loaded cells

*Orpk* cilia (-) cells were cultured on black walled, clear bottom 96-well plate to become confluent and were serum-starved overnight. Cells were then loaded with Fura-2 for one hr in a low [Na^+^] control buffer solution containing (in mM): 10 NaCl, 4.8 KCl, 10 D-Glucose, 10 HEPES, 1.8 MgCL_2_, 1 CaCl_2_, 1 Probenecid, 100 NMDG·Cl. Cells were rinsed in this same solution minus the Probenecid but containing 10 ng/mL EGF for 20 min. Then the cells were exposed to this same solution but containing 0.5 mM Mn^2+^. Fluorescence intensity was measured in plates ~3 mins after addition of Mn^2+^ to each of the 96 wells (excitation wavelength of 359 nm, isosbestic-Ca^2+^ insensitive wavelength, with an emission wavelength of 510 nm, at 37°C). This measurement of fluorescence intensity was performed because it was not possible to measure the real time decay in Fura 2 fluorescence in a 96 well plate. In preliminary experiments we found that, in response to the addition of Mn^2+^, there was a rapid decrease in Fura 2 fluorescence that stabilized after ~ 1 min compared to the very little change in Fura 2 fluorescence in the absence of Mn^2+^.

### TRPP2 and TRPV4 genes silencing

Cells were cultured under permissive conditions as described (refer to above) using 100 mm tissue culture dishes. After cells became 70% confluent, TRPP2-specific and TRPV4-specific short hairpin RNA (shRNA) constructs were transfected using lipofectamine (Invitragen, USA; refer to Table S2 for the sequences of shRNA constructs). Because all shRNA constructs contain a puromycin resistance gene, the 2^nd^ day following transfection, cells were continuously cultured in the presence of 10 μg/mL puromycin. Seven days after transfection, cells were transferred onto permeable support attached to *Snapwell* inserts and were cultured for 10 to 14 days in the presence of 10 μg/mL puromycin.

### Co-immunoprecipitation of TRPP2 with TRPV4

Cells were plated in permeable supports and grown to confluence. For EGF treatment, cells were serum starved overnight followed by incubation with 10 ng/mL of EGF for 30 min. Cells were rinsed with ice cold PBS and lysed in ice cold Triton buffer and 1 mM PMSF, and 100x dilution of HALT phosphatase inhibitors (Pierce) for 30 min on ice. Lysates were spun at 17,000 x g for 15 min to pellet insoluble cellular material and supernatant was pre-cleared by incubation with 30 µL protein A/G Sepharose beads for 30 min at 4^o^C. After a brief centrifugation, the supernatant was removed and aliquots of proteins (500 µg) were incubated with 4 µg of anti-TRPP2 antibody (UAB P30 Developed Antibody based on the YCC2 antibody of Somlo) or anti-TRPV4 antibody (Santa Cruz #47527) overnight at 4^o^C. Immunoprecipitates were captured with 40 µL of protein G beads (Santa Cruz) at 4^o^C for one hr. Samples were centrifuged, washed three times with 1 mL of Triton lysis buffer, and eluted from the beads using 2x Laemli sample buffer. Samples were subsequently analyzed by SDS-PAGE using 3-8% Tris-acetate gels (Invitrogen, USA) and transferred to PVDF membrane. Blots were probed with 1:500 dilutions of anti-TRPV4 antibody, or with 1:1000 dilution of TRPP2 antibody overnight at 4^o^C. After incubating with appropriate horseradish peroxidase-conjugated secondary antibody (1:15,000, Jackson ImmunoResearch) and blots were developed using SuperSignal West Dura extended duration substrate (Pierce) and CL-XPosure Film (Pierce).

### Western blot analysis

The expression level of EGFR, ERK, phosphor-ERK (p-ERK) and the efficiency of gene silencing by shRNAs were evaluated by western blotting analysis. Cells were harvested and lysed in 4% SDS in PBS. Samples were separated by SDS/PAGE and transferred to PVDF membrane. Membranes were blocked in 5% wt/vol milk followed by incubation with primary antibodies against EGFR (Upstate #06-129, diluted at 1:1000), ERK (Cell signaling #9107, diluted at 1:1000), p-ERK (Cell signaling #9101S, diluted at 1:1000); the anti-TRPV4 ployclonal antibody with 1:500 dilution [15], and TRPP2 (diluted at 1:200), β-actin (Sigma #A5441, 1:10000), respectively. After incubating with horseradish peroxidase-conjugated, donkey anti-sheep HRP (Jackson ImmunoResearch #713-035-003, diluted at 1:10000), goat anti-mouse HRP (Jackson ImmunoResearch #115-035-003, diluted at 1:30000), and goat anti-rabbit HRP (Jackson immunoresearch # 111-035-003, diluted at 1:5000) blots were developed using SuperSignal West Dura extended duration substrate (Pierce) and CL-XPosure Film (Pierce).

### Quantitative real time RT-PCR analysis

This assay was used to evaluate the gene silencing efficiency by shRNAs transfection. Total RNA was prepared from *orpk* cilia (-) cells grown in permeable supports using Trizol reagent (Invitrogen) according to the manufacturer’s instructions. The extracted total RNA was subjected to DNase treatment using the Turbo DNA-free kit (Ambion). cDNAs were generated from 2 µg of total RNA using Superscript III First-Strand Synthesis kit for qRT-PCR (Invitrogen) and random hexamer primer, according to the recommendations of the manufacturer. Quantitative real-time RT-PCR was performed using specific validated primer pairs for mouse TRPV4 (NM_022017), TRPP2 (PkD2) (NM_008861.3) and GAPDH (glyceraldehyde-3-phosphate dehydrogenase) (NM_008084.2) (SABioscience). Negative control reactions lacking reverse transcriptase as well as no template reactions were used to rule out the possibility of interference from genomic DNA contamination during the qPCR reactions. Each primer pair was analyzed in duplicate for each cDNA sample. Reactions for qRT-PCR were set up in a 96-well plate format in a 25 µL reaction volume containing 12.5 µL of RT^2^ SYBR Green/Fluorescein qPCR Master Mix (SABioscience), 200 ng cDNA and each primer at 400 nM final concentration. The qPCR reactions were performed in a MyiQ thermal cycler (Bio-Rad) with the following conditions: 95^o^C for 10 min followed by 40 cycles of 95^o^C for 15 sec and 60^o^C for one min. We utilized the ΔΔ*Ct* (*threshold cycle*) method to determine the relative TRPV4 and TRPP2 transcript levels in a given cDNA sample as recommended by the manufacturer of the qRT-PCR assays. Analysis of variance (ANOVA) followed by Bonferroni’s multiple comparison test was performed on the differences between the Ct(GAPDH) and Ct(TRPV4) or Ct(PC2) (ΔCt) to determine the statistical significance of variations in the transcript levels of TRPV4 and TRPP2. Percent changes between the control and shRNA treated samples were calculated by the following formula: % of control=100x2^(ΔCt^_(Co)_^-ΔCt^_(shRNA)_^)^.

### Immunofluorescence staining

Studies were performed using confocal microscopy. *Orpk* cilia (+) and cilia (-) cells, grown on permeable supports for 10-14 days, were washed with PBS and fixed with 4% paraformaldehyde in PBS at room temperature. Cells were permeabilized with 0.2% Triton X-100 and subsequently washed with PBS. Blocking of nonspecific binding was accomplished by incubating in 1% BSA in PBS. Cells were incubated for one hr with primary antibody against TRPV4 diluted at 1:200 [15]; and primary antibody against TRPP2 diluted at 1:800 and subsequently incubated with Alexa Fluor 488-conjugated and Alexa Fluor 594-conjugated secondary antibodies (1:1000, Molecular Probes, Eugene, OR) for 45 min. Cells were washed and mounted with glycerol media. Slides were imaged with a Leica SP5 AOBS scanning laser confocal microscope in xz reconstruction mode using a 63x Plan Apochromat 1.40 oil immersion objective. Identical acquisition settings were used on all images.

### Cell proliferation assay

Cells were serum starved for overnight prior to the assay. Then 2,000 cells were seeded in each well of a 96-well plate. Twelve hrs after seeding the cells, either 100 nM AG1478, or 20 μM PD98059, or 10 μM BAPTA/AM were added into designated wells, followed two hours later by 10 ng/mL EGF added into each well. Additional 10 ng/mL EGF was added each day until the proliferation assay was performed. Cells were harvested at 0, 24, 48, 72, 96 hours (twelve hrs after seeding the cells was designated as zero hour) washed with serum-free medium and stored at -80°C. Cell proliferation rate was obtained by using a well established method, CyQUANT cell proliferation assay kit from Molecular Probes (Invitrogen) [16–18]. The rational for the CyQUANT assay is the use of a proprietary green fluorescent dye (CyQUANT GR dye) that exhibits strong fluorescence enhancement when bound to cellular nucleic acids. The detected fraction of nucleic acid (DNA) is proportional to total cell number and was used as an index of cell proliferation. Briefly, frozen cells were thawed and lysed with a buffer containing the CyQUANT GR dye. Fluorescence was measured using Cytofluor II fluorescence multi-well plate reader (Molecular Devices, Sunnyvale, CA, USA) at an excitation wavelength of 485 nm and emission wavelength of 530 nm. The results presented are the mean of six separate experiments.

### Statistics

Data are reported as mean values ± SD for *n* observations. Statistical analysis was performed by using SigmaPlot and SigmaStat software (Jandel Scientific, CA. USA). Data were compared using paired or unpaired Student’s *t* test. Analysis of variance was used for multiple comparisons. Differences were considered statistically when *p* < 0.05.

## Results

### EGFR tyrosine kinase- and MAPK-dependent signaling regulates a spontaneous channel at the apical membrane

**Figure 1:**
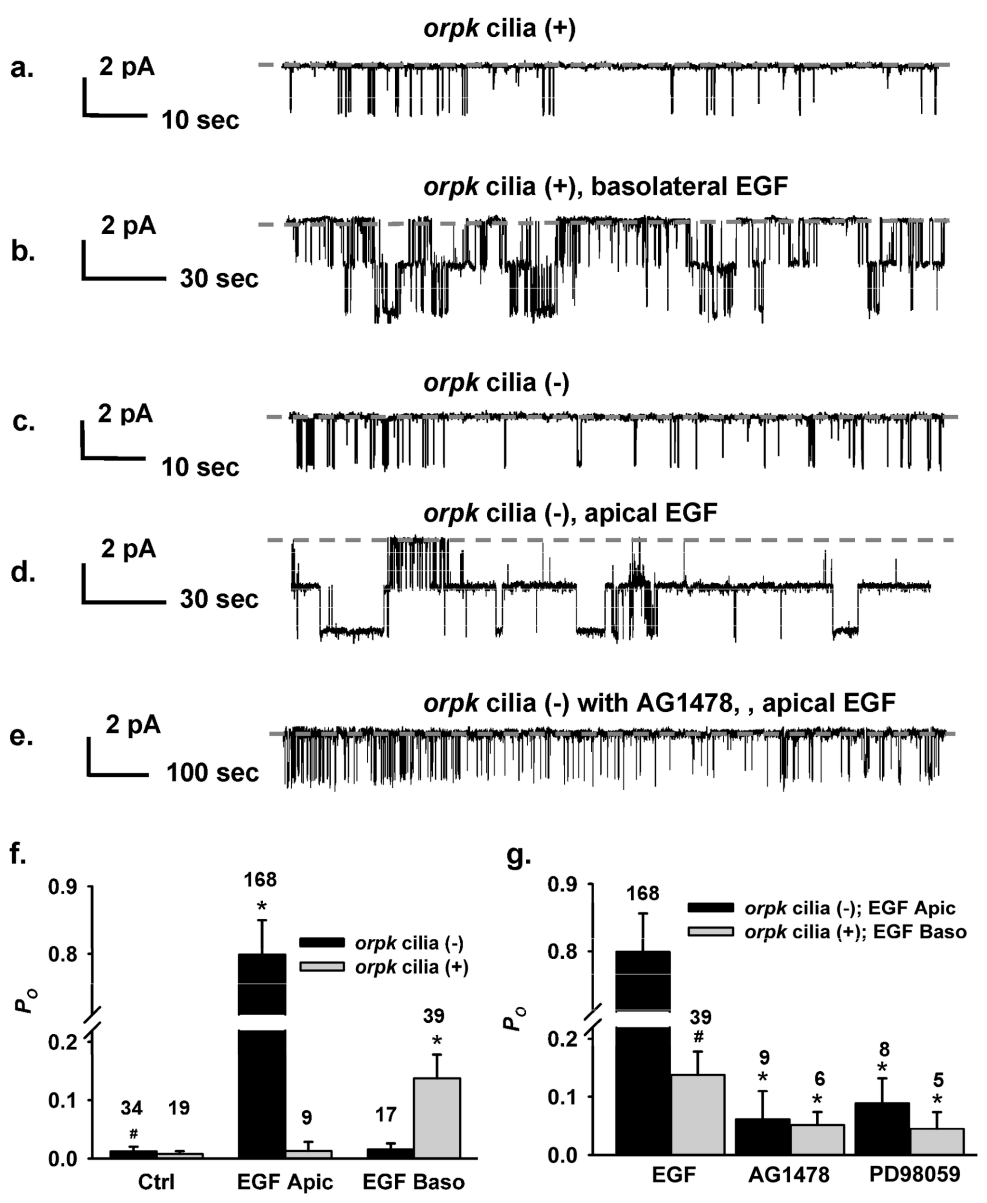
EGF regulates an apical 23-pS cation channel in both cilia (+) and cilia (-) cells. (**a**–**e**) The representative single-channel traces were recorded from cilia (+) and cilia (-) cell using excised inside-out patch mode, respectively. 10 ng/mL EGF was added to the apical or the basolateral side for 5 to 10 min before recordings. The dashed gray lines indicate closed levels of current. Representative single-channel traces were recorded from cilia (+) (**a** & **b**) and cilia (-) cells (**c** & **d**) in the presence of basolateral or apical EGF, respectively. (**e**) A representative single-channel current was recorded from cilia (-) cell that was pretreated with 100 nM AG1478 for 30 min followed by apical administration of EGF. (**f**) Summary of *P*_*O*_ values obtained under different experimental conditions in both cell types. * indicates significant difference from controls and ^#^ indicates significant difference between two cell types under control conditions; where Ctrl, EGF Apic and EGF Baso represent control, EFG applied at the apical and at the basolateral membrane, respectively. (**g**) Summary of the effects of tyrosine kinase inhibitor and MAPK inhibitor on EGF stimulated channel activity. * indicates the channel activity was significantly blunted by tyrosine kinase and MAPK inhibitors and ^#^ indicates EGF-induced channel activity is significantly different between the two cell types. Inserted numbers in Figure 1f & 1g represent *n* recordings under indicated experimental conditions.

Using inside-out patch-clamp analysis we detected a spontaneous single-channel current with a very low open probability (*P*_*O*_) in both cilia (+) and cilia (-) cells (0.008 ± 0.005 in cilia (+) cells vs. 0.013 ± 0.008 in cilia (-) cells) (Figure **1a, 1c** **& 1f**). Beyond the significantly greater *P*_*O*_ in cilia (-) versus cilia (+) cells (Figure **1f**), the rate of occurrence of this channel was ~20% higher in cilia (-) cells compared to cilia (+) cells (46.3% vs. 25.6%).

To test the hypothesis that EGF may regulate this channel, 10 ng/mL EGF was added to either the apical or basolateral solutions of cells grown on permeable supports for 5-10 min prior to patch-clamp analysis. The rationale for adding EGF to apical or basolateral sides is that EGFR has been reported to be mislocalized to the apical membrane in PKD. We verified that there was EGFR mislocalization in cilia (-) cells (data not shown). In cilia (+) cells, *P*_*O*_ of this channel was increased by ~17-fold with addition of EGF added to the basolateral membrane (Figure **1a, 1b** **& 1f**). In cilia (-) cells, *P*_*O*_ of this channel was increased ~64-fold with addition of EGF added to the apical membrane (Figure **1c, 1d** **& 1f**). Interestingly, when additional EGF applied to the apical membrane in cilia (+) cells or to the basolateral membrane of cilia (-) cells, it failed to increase *P*_*O*_ of this channel (raw data not shown; **1f**). The regulatory effect of EGF on this apical channel was markedly attenuated by pre-incubation with 100 nM AG1478, a tyrosine kinase inhibitor, and 20 µM PD98059, a MAP kinase inhibitor (Figure **1e & 1g**) with a much greater efficacy in cilia (-) cells, suggesting that EGF-dependent signaling contributes to the regulation of this channel.

**Figure 2:**
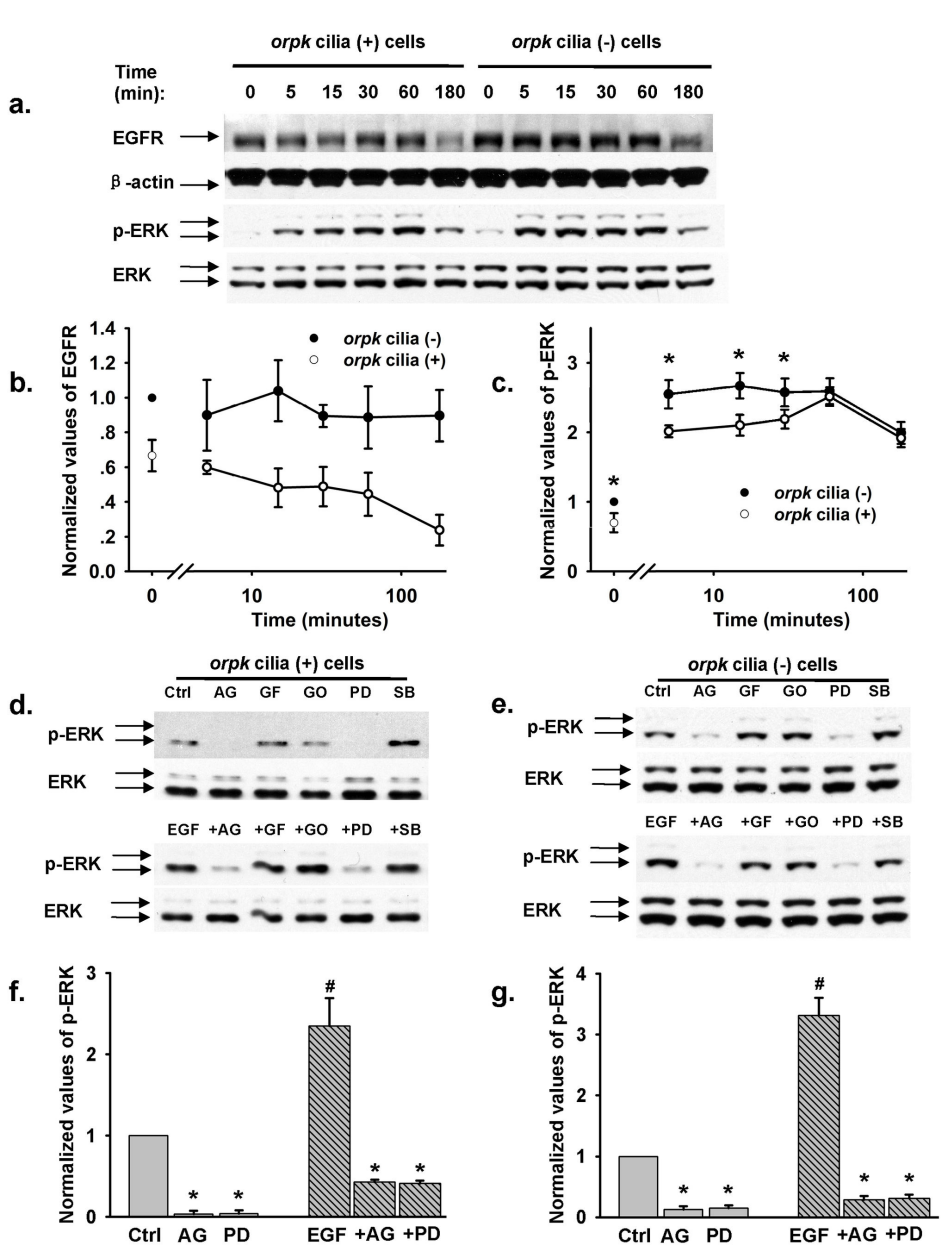
Cilia (-) cells has a much higher activity of EGFR and p-ERK than cilia (+) cells. (**a**) The representative western blots showing the expression of EGFR, ERK, and p-ERK before and after 10 ng/mL EGF treatment from both apical and basolateral sides in both cell types for the indicated time. (**b**) Summary of EGFR expression levels, all data points were normalized to the value obtained from cilia (-) cells under control condition, i.e. no EGF simulation (n = 5 for each data point). (**c**) Summary of p-ERK expression levels, all data points were normalized to the value obtained from cilia (-) cells under control condition, i.e. no EGF simulation (n = 6 for each data point). * indicates p-ERK expression level of cilia (-) cells is significantly greater than that of cilia (+) cells. (**d** & **e**) The representative western blots showing the expression ERK and p-ERK with and without stimulation by 10 ng/mL EGF from both apical and basolateral sides in both cell types for 10 min, in the presence of variety of inhibitors. Where Crtl indicates control and AG, GF, GO, PD, and SB represent AG1478, GF109203, GO6976, PD98059, and SB202190, respectively. Cells were preincubated with EGF for 60 min, respectively. (**f** & **g**) Summary of p-ERK expression levels, all data points were normalized to the value obtained under control condition, i.e. no EGF simulation (n = 5 for each data point). * indicates the values were dramatically less than those of control and EGF stimulation. ^#^ indicates p-ERK expression level is significantly greater compared to control.

Shown in Figure **2a** (upper panel) and Figure **2b** are EGFR expression levels at various time points before and after addition of 10 ng/mL EGF. There was a greater abundance of EGFR in cilia (-) cells compared to cilia (+) cells in the absence of EGF. In the presence of EGF, there was a time-dependent decay of EGFR protein in cilia (+) cells perhaps suggesting internalization and degradation of this receptor, which is consistent with known ligand-EGFR interactions [19,20]. In cilia (-) cells, there was no significant ligand-induced loss of EGFR activity, at least over a span of three hours. EGF did not alter the abundance of ERK in either cell type (Figure **2a**). However, without stimulation by EGF, there was a slight, but significant, increase in abundance of p-ERK in cilia (-) cells compared to cilia (+) cells ; in the presence of EGF for 5-30 min, the abundance of p-ERK was dramatically increased in both cell types, albeit to a much greater extent in cilia (-) cells (Figure **2a & 2c**). Interestingly, p-ERK activity in cilia (+) and cilia (-) cells was increased to the same levels one hr after stimulation with EGF (Figure **2a & 2c**). As shown in Figure **2d–2g** the abundance of p-ERK, with or without stimulation of EGF, was dramatically attenuated by 100 nM AG1478 and 20 µM PD98059 in both cell types. Whereas 3 µM GF109203, a selective protein kinase C (PKC) inhibitor, 3 µM Go 6976, a PKC α/β inhibitor, and 10 µM SB202190, a P38 MAPK inhibitor did not affect the abundance of ERK or p-ERK (Figure **2d & 2e**).

### The 23-pS channel functions as a non-selective divalent cation-permeable channel at the apical membrane and EGF promotes Ca^2+^ influx in cilia (-) cells via this channel

**Figure 3:**
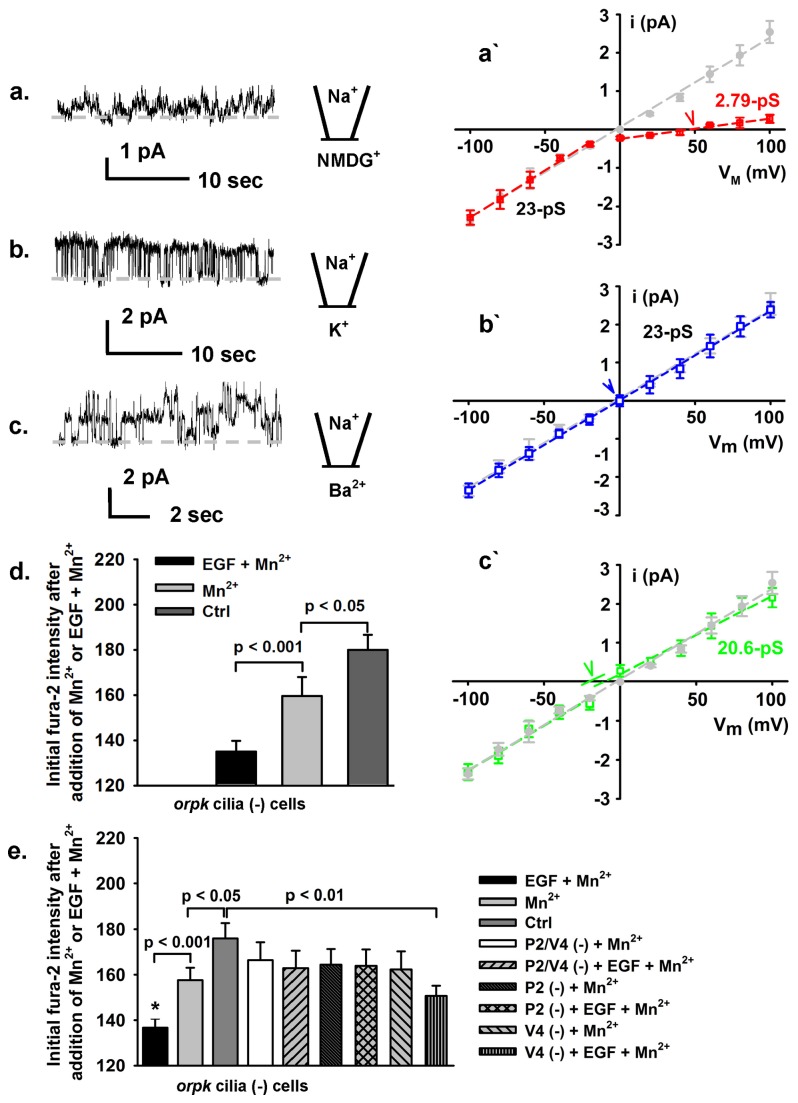
~23-pS channel permeates divalent cation and mediates EGF-induced Ca^2+^ influx. Cilia (-) cells were pretreated with 10 ng/mL EGF at the apical membrane prior to recordings. (**a**–**c**) The representative traces were recorded using excised inside-out patch mode at V_M_ = 100 mV from cilia (-) cells and the dashed gray lines indicate closed current level. (**a**`–**c**`) The gray dashed lines indicate the fitted single-channel conductance (23.4 ± 0.76 pS) of the currents recorded under control condition (representative single-channel traces are shown in Figure S1; n = 13-21 for different data points). (**a** & **a**`) This representative recording contained at least four active channels. While the single-channel conductance of inward currents remained the same as control the single-channel conductance of outward currents was dramatically reduced to 2.79 ± 0.33 pS (n = 9-14 for different data points) by substitution of intracellular Na^+^ with equimolar NMDG^+^ and the reversal potential shifted to depolarization potentials (the red arrow head). (**b** & **b**`) The representative trace was recorded in the presence of equimolar intracellular K^+^ to extracellular Na^+^, which affected neither the single-channel conductance (the blue dashed line) nor the reversal potential (the blue arrow head). (**c** & **c**`) The representative trace was recorded in the presence of equimolar intracellular Ba^2+^ to extracellular Na^+^. Replacement of Na^+^ by Ba^2+^ did not alter the single-channel conductance of inward current, but altered the single-channel conductance of outward current (the green dashed line) and the reversal potential of the currents (where the green arrow head indicates the reversal potential that was corrected for the junction potential). (**d** & **e**) Manganese quenching assays demonstrate that EGF promotes Ca^2+^ entry in cilia (-) cells, but not in cilia (+) cells (data not shown); while double knockdown of TRPP2\TRPV4 channels or TRPV4, the effect of EGF on Ca^2+^ entry was abolished (n = 5); while knock down of TRPV4, EGF also promotes Ca^2+^ entry, albeit with less extent compared to wild-type cilia (-) cells (n = 5). * indicates significant difference compared to TRPV4 knocked down cilia (-) cells while stimulated with EGF.

Using inside-out patch configuration we identified a channel with a linear i-V relationship at the apical membrane, having an identical single-channel conductance (~23-pS; the gray dashed fit line Figure **3a`**) in cilia (+) cells (data not shown) and in cilia (-) cells (representative single-channel traces shown in Figure **S1**). When intracellular Na^+^ was substituted with equimolar n-methyl-D-glutamine (NMDG)^+^, reversal potential shifted toward depolarizing potentials (the red arrow head in Figure **3a`**). While the inward current was the same compared to control (the red dashed fit line for inward currents of Figure **3a`**), there were small outward currents remained in the patches (Figure **3a** & Figure **3a`**) with a single-channel conductance of ~2.8-pS (the red dashed fit line in Figure **3a`**); this small outward currents detected at high depolarizing potentials is likely carried by the small amounts divalent cations remaining in the bath solution (5 mM K^+^, 1.5 mM Ca^2+^). Substitution with equimolar K^+^ for Na^+^ did not alter either the single-channel conductance or the reversal potential (the blue dashed fit line for outward currents and the blue arrow head, Figure **3b & 3b`**). In the presence of intracellular Ba^2+^ the single-channel conductance of inward current was not altered compared to control (the green dashed fit line for inward currents in Figure **3c`**); however, the single-channel conductance of outward current was decreased by ~10% (~20.6-pS, the green dashed fit line for outward current in Figure **3c`**) compared to control and the reversal potential shifted toward hyperpolarization direction (the green arrow head; Figure **3c**, Figure **3c`**). The relative permeability ratio for P_Ba_^2+^/P_Na_^+^ was 1.97 ± 0.41, suggesting that this channel has a greater permeability to divalent cations. This channel equally conducts Ba^2+^ in the opposite direction from extracellular to the intracellular side; the relative permeability ratio was P_Ba_^2+^/P_Na_^+^ = 1.89 ± 0.37 (raw data not shown). We also performed the ion selectivity experiment in cilia (+) cells and the results were identical to those in cilia (-) cells (data not shown). To rule out the possibility of this channel is mediated by TRPM4 or epithelial sodium channel (ENaC), ATP or amiloride was added to the both solution. The data show that neither 2 mM ATP (Figure **S2a**) nor 10 µM amiloride (Figure **S2b**) inhibited this ~23-pS channel.

Mn^2+^ quenches Fura-2 fluorescence when measured at the Ca^2+^ insensitive excitation wavelength of 359 nm and can be used as an index of Ca^2+^ entry. As shown in Figure **3d**, cilia (-) cells exposed to apical Mn^2+^, in the absence of EGF, demonstrated a slight, but significant, decrease in Fura-2 fluorescence, compared to Fura-2 loaded non-treated control cells; whereas apical EGF addition greatly diminished Mn^2+^-induced decrease in Fura-2 fluorescence. In TRPP2 knocked down, or TRPP2 & TRPV4 double knocked down cilia (-) cells (for knocking down efficiency, please refer to below), apical EGF addition dramatically diminished Mn^2+^-induced decrease in Fura-2 fluorescence compared control cells (Figure **3e**). However, in TRPV4 knockdown cilia (-) cells (for knocked down efficiency, please refer to below), additional apical EGF induced a significant decrease in Fura-2 fluorescence; however, the degree of decrease in Fura-2 fluorescence in TRPV4 knock down cells was significantly less than that in normal cilia (-) cells. This could be explained by the fact that after knock down of TRPV4 there were more “free” TRPP2 channels (Table **1**) available to be activated by EGF [11]. As expected, cilia (+) cells did not show Mn^2+^-dependent Fura-2 quenching with addition of apical EGF (data not shown).

**Table 1:** The *orpk* cilia (-) cells were used to identify the molecular identity of EGF stimulated ~23-pS channel.

***Typeofknockingdown***	***23-pS%***	***TRPV4- like %***	***TRPP2-like%***	***Empty%***	***(n)***
TRPV4	18.18	0	16.36	65.16	110
TRPP2	9.52	7.79	0	82.68	63
TRPV4/TRPP2	5.3	3.27	1.63	89.8	61
Wild-type	59.57	0	0.019	40.411	168

% represents the appearance rate of certain type of channels under given experimental condition in *n* patches. Wild-type indicates the cells were not transfected with shRNA specific to TRPP2 or TRPV4. Empty means neither ~23-pS channels, nor TRPV2- and TRPP2-like channels were observed in the patches. *n* represents the total number of patches for indicated condition.

### TRPP2 and TRPV4 assemble to form the 23-pS channel complex at the apical membrane

**Figure 4:**
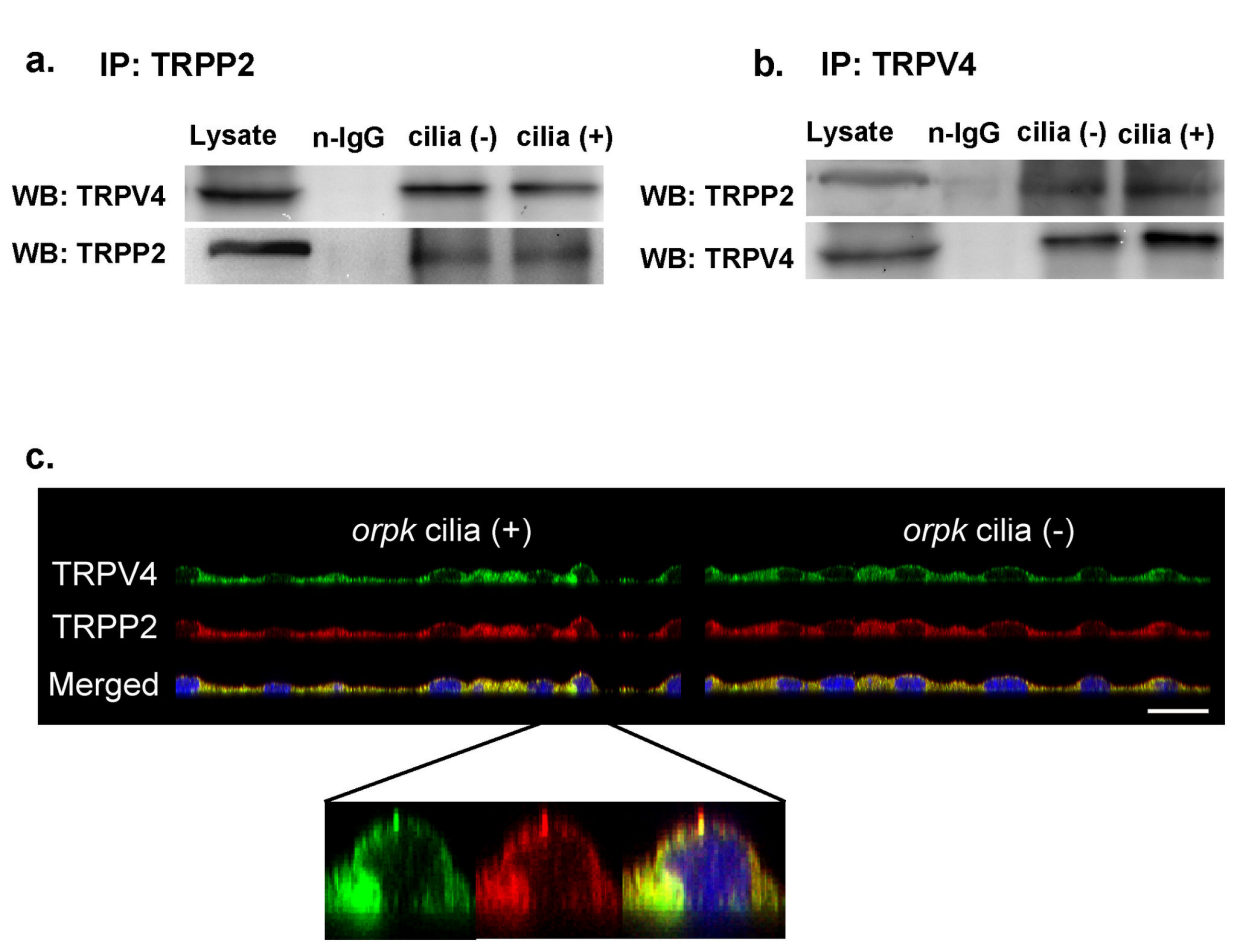
Co-IP and immunofluorescence staining demonstrating formation of a TRPP2\TRPV4 complex. (**a** & **b**) Cells were immunoprecipitated with anti-TRPP2 and anti-TRPV4 antibodies, respectively. Co-precipitated complexes were probed by TRPV4 and TRPP2 antibodies, respectively. Cell lysates and n-IgG were served as controls. (**c**) Representative reconstructed side view (xz) immunofluorescence images of cilia (+) and cilia (-) cell monolayers fixed and labeled with antibodies raised against TRPV4 (green) and TRPP2 (red). Yellow color on the merged pseudocolor images at the bottom indicates colocalization of TRPV4 and TRPP2. The length of the bar in the bottom right is 30 µm. The inset demonstrates a single cell with a prominent apical cilium. Although TRPP2 appears to be more abundant at the tip of cilium, this is the result of a shift in the position of the red and green images in the z plane due to a slight chromatic aberration correction error in the z-plane.

We tested the hypothesis that this channel might be a heteromeric channel complex comprised of different TRP proteins. RT-PCR was used to identify TRP channels in cilia (+) and cilia (-) cells (refer to Table **S1** for primers used) and TRPV4, TRPP1, TRPP2, TRPM4, TRPM6, TRPC1 and TRPC2 channels were detected in both cell types (Figure **S3a**). However, the biophysical properties of this 23-pS channel are distinct from the individual channel mentioned above. TRPM4 can be inhibited by ATP and does not permeate to divalent cations [21]; TRPM6 has a single-channel conductance ~83-pS [22] and a much higher permeability to divalent cations [16]; TRPV5 and TRPV6 channels are characterized by voltage-dependent opening at negative potentials and extremely strong inward rectification with single-channel conductance of 75-90-pS and 40-70-pS, respectively [23,24]; TRPC1 channels are non-selective between Ba^2+^ and Na^+^ with a single-channel conductance of ~16-pS [23]; TRPC2 channels have significant higher permeability to divalent cation compared to the 23-pS channel and a single-channel conductance of ~42-pS [24]; TRPP2 channel has a single-channel conductance of 40-177-pS and can be blocked by amiloride [4]; the heteromeric TRPP2/TRPC1 channel complex functions as GPCR-activated channel with a single-channel conductance of ~40-pS [3–5]; finally, TRPV4 channels exhibits strong outward rectification with single-channel conductance of ~90-pS [25,26]. Therefore, based on the results obtained by Mn^2+^ quenching assays (Figure **3d & 3e**) and the work of Kötten et al. and Stewart et al. [6,7] we focused on a potential interaction between TRPP2 and TRPV4. As shown in Figure **4a & 4b**, TRPV4 and TRPP2 were pulled down with a TRPP2 antibody and a TRPV4 antibody, respectively, in both cell types using co-immunoprecipitation (co-IP) followed by western blot analysis. The data demonstrate that TRPP2 & TRPV4 proteins physically associate in both cell types. Furthermore, this same association between TRPP2 and TRPV4 was found in inner medullary collecting duct (IMCD) cells (Figure **S3b**). As shown in Figure **4c**, immunofluorescence stainings also support the notion that TRPV4 and TRPP2 present and co-localize in the apical and lateral membranes, as well as in cilia.

**Figure 5:**
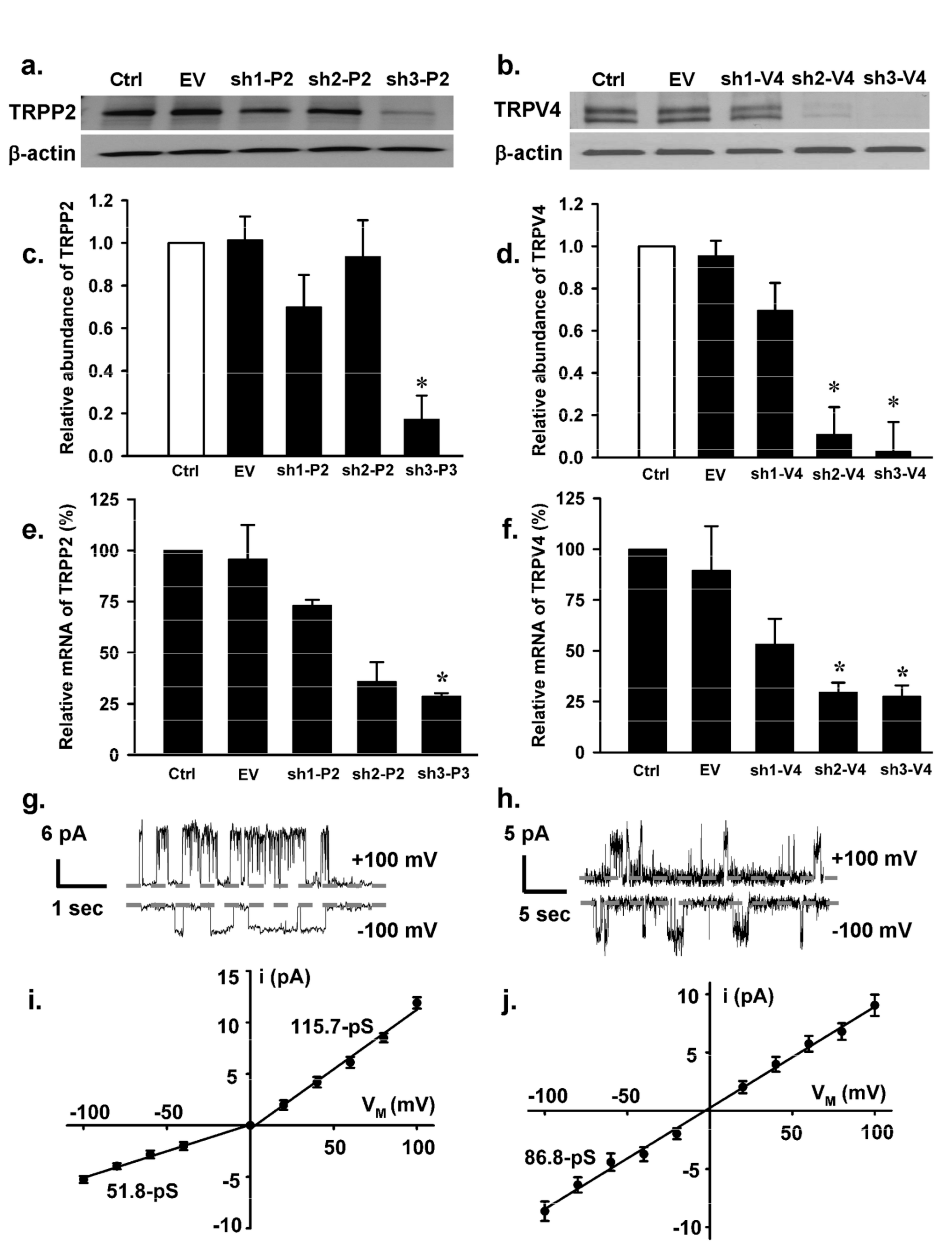
Effect of gene silencing on the 23-pS single-channel currents. (**a**–**b**) Western blots showing knockdown of TRPP2 (left) and TRPV4 (right) by TRPP2- and TRPV4-specific shRNAs. (**c**–**f**) Summary of the data obtained from western blots (**c & d**; n = 5) and quantitative real-time PCR analysis (**e & f**; n = 3). Ctrl, EV, sh1, sh2 and sh3 represent non-tranfected (control), empty vector, shRNA1, shRNA2 and shRNA3 transfected cells, respectively. P2 and V4 indicate that the shRNAs were specific to TRPP2 and TRPV4, respectively. * indicates significant difference from control and empty vector transfected group. (**g** & **h**) Sample single-channel traces were recorded from cilia (-) cells expressing shRNA3s specific to TRPP2 and TRPV4, using excised inside-out patch configuration. (**i** & **j**) Summary of i-V plots constructed from the single-channel recordings as shown (**g**) and (**h**) demonstrating altered biophysical properties of the channels. Knockdown TRPP2 and TRPV4 resulted in the appearance of TRPV4-like channels (**i**) with a single-channel conductance of 115.7 ± 1.98 pS for outward and 51.8 ± 0.91 pS for inward currents, respectively and TRPP2-like currents with a single-channel conductance of 86.8 ± 1.78 pS (n = 5-9 for different data points).

**Figure 6:**
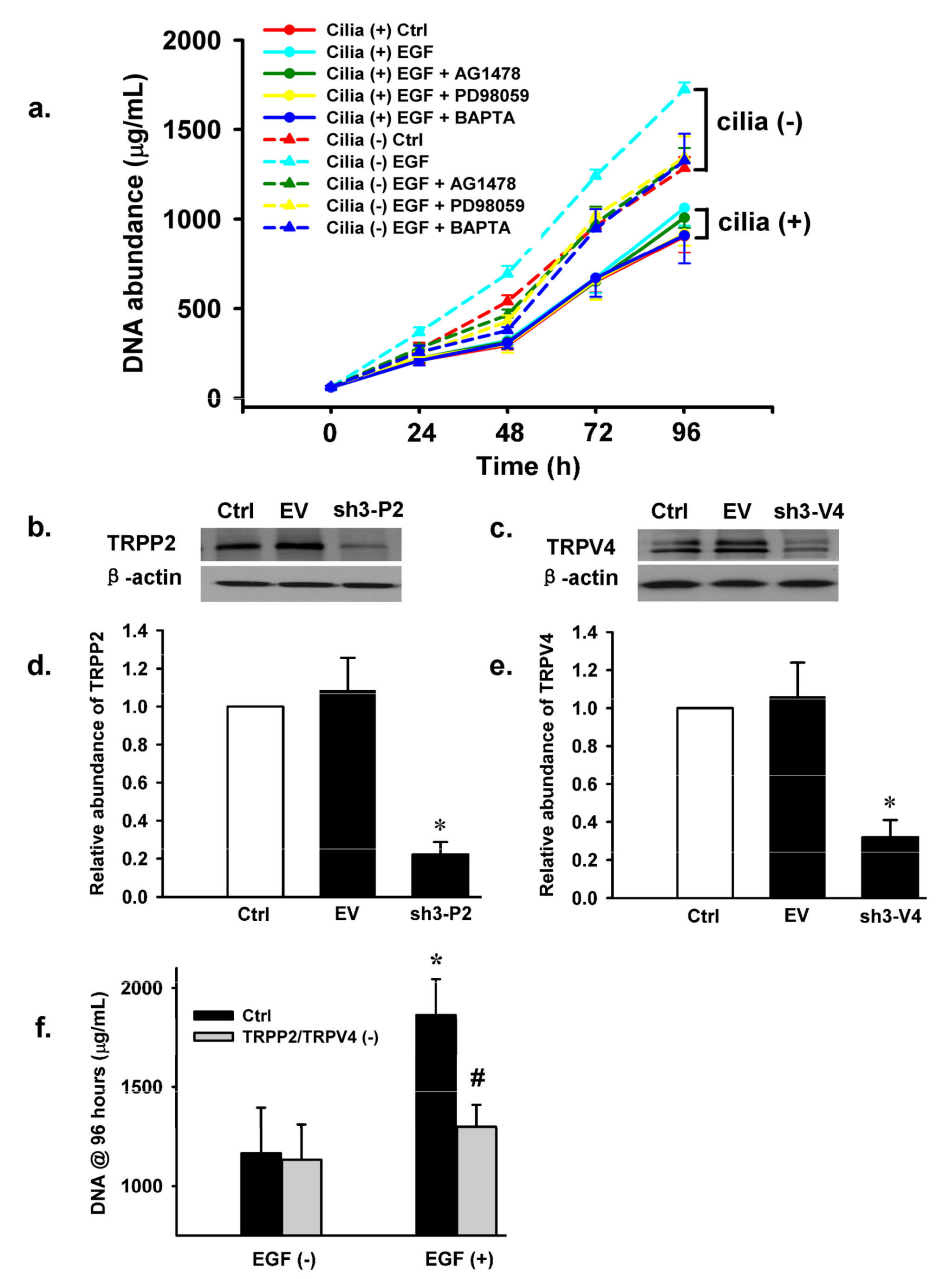
EGF-induced activation of TRPP2\TRPV4 channels mediates hyperproliferation of cilia (-) cells. (**a**) Plot shows the rate of cell proliferation. Under control conditions, cell proliferation rate was significantly higher in cilia (-) cells (the red dashed line) than in cilia (+) cells (the red solid line). EGF significantly enhanced the rate of cell proliferation in cilia (-) cells but not in cilia (+) cells. The effect of EGF on cell proliferation was abolished by AG1478, PD98059 and BAPTA in cilia (-) cells (n = 5 for each condition). (**b** & **c**) Western blots confirm the effectiveness of knockdown of both TRPP2 and TRPV4, where Ctrl, EV, sh3-P2\sh3-V4 represent non-tranfected (control), cells co-transfectd with empty vector, sh3-P2\sh3-V4 specific to TRPP2 and TRPV4, respectively. (**d** & **e**) Summary of the results obtained from western blots analysis; both TRPP2 and TRPV4 protein expression levels were reduced by ~75-80%. (**f**) Cell proliferation assays were performed at 96 hour time point after seeding the cells in the presence or in the absence of EGF. Plot shows that the rate of EGF-induced proliferation in cilia (-) cells was significantly reduced by knockdown TRPP2\TRPV4 channels. KO indicates knockdown, * indicates that under control condition the rate of cell proliferation was significantly higher in the presence of EGF. # indicates a significant difference compared to control.

shRNA technologies were used to further assess the interactions between TRPV4 and TRPP2. We first evaluated the effectiveness of gene silencing with several different shRNA constructs using Western blot (Figure **5a-5d**) and quantitative real-time PCR analysis (Figure **5e & 5f**). The construct sh3-P2 was most effective in knocking down TRPP2 while both sh2-V4 and sh3-V4 constructs were effective in knocking down TRPV4 (Figure **5a–f**). Cells containing these constructs were then subjected to patch-clamp analysis. Knockdown of TRPP2 resulted in the appearance of an outwardly rectifying channel, with a single-channel conductance of 51.8-pS for inward current and 115.7-pS for outward currents, (Figure **5g & 5i**) which is very similar to what has been reported for TRPV4 [25,26]. With TRPV4 knockdown (Figure **5h & 5j**) we were able to detect a channel with a single-channel conductance very similar to that reported for TRPP2 [27]. Furthermore, in either TRPP2 or TRPV4 knockdown cells, the presence of the EGF-activated ~23-pS channel was greatly reduced in TRPV4 knockdown cells (~18%) and ~10% in TRPP2 knockdown cells vs. ~59.6% in control cells) (Table **1**). In addition we performed double knockdown of TRPP2 and TRPV4 experiments in cilia (-) cells (Western blots in Figure **6b–e** show the effectiveness of knock down). With reduced expression level of TRPP2 and TRPV4 the appearance rate of EGF-induced 23-pS channel was dramatically decreased compared to non-transfected cells (5.3% vs. ~59.6%; Table **1**).

### EGF-induced aberrant activation of TRPP2\TRPV4 channels promotes hyperproliferation of cilia (-) cells in vitro

As shown in Figure 6a, under control conditions cilia (-) cells exhibited a greater rate of cell proliferation. Incubation of cilia (+) cells with apical EGF did not alter the rate of cell proliferation, presumably because these cells were grown in cell culture dishes and EGF did not have access to the receptor. Importantly, cell proliferation rate of cilia (-) cells was accelerated significantly by addition of EGF. This enhanced cell proliferation with EGF was greatly attenuated by AG1478, PD98059, and BAPTA/AM (Ca^2+^ chelator), respectively. To determine if the TRPP2\TRPV4 channel is involved in this pathway gene-silencing technology was employed by co-transfecting cilia (-) cells with constructs sh3-P2 (to knockdown TRPP2) and sh3-V4 (to knockdown TRPV4). As shown in Figure **6b-6e** western blots demonstrate that both proteins were effectively knocked down. As expected, proliferation rate was significantly greater in cilia (-) cells that were incubated with EGF (Figure **6f**). This EGF dependent increase in cell proliferation was significantly decreased in TRPP2 and TRPV4 knock down cilia (-) cells. These results strongly suggest that EGFR-dependent signaling activates the TRPP2\TRPV4 channel complex that leads to an increase in [Ca^2+^]_i_ and thereby contributes to enhanced proliferation in cilia (-) cells.

## Discussion

The current study demonstrates that endogenous TRPP2 and TRPV4 assemble to form a non-selective divalent cation-permeable cation channel, with a single-channel conductance of ~23-pS at the apical membrane of *orpk* mouse collecting duct cells. Furthermore, EGF stimulates this TRPP2\TRPV4 channel through downstream signaling of ligand-EGFR interactions. Our data suggest that EGF promotes calcium influx through this channel and subsequently enhances cell proliferation rate in *orpk* cilia (-) cells suggesting that this pathway may play a role in polycystic kidney disease.

### Endogenous TRPP2 and TRPV4 assemble to form a non-selective calcium permeable channel complex

Previous work has reported that protein–protein interactions occur within specific family members of TRP channels; in this regard, members of TRPC appear to be particularly promiscuous [27]. These protein–protein interactions, however, also occur between members of different TRP families, where association between various members of the TRPC and TRPV sub-families have been particularly well studied [27]. Most recently, there have been several reports describing interactions of TRPP2 with other members of the TRP family [3–7]. Bai and co-workers have demonstrated, using cultured rat sympathetic neurons, over expression of TRPP1\TRPP2, TRPP2, TRPC1 and TRPP2\TRPC1, that the TRPP2\TRPC1 complex is a GPCR-activated channel [3]. These authors have found that this channel exhibits a pattern of single-channel conductance (40-pS), amiloride sensitivity, and ion permeability distinct from that of individual TRPP1\TRPP2 (142-pS) or TRPC1 (16-pS) channel. Subsequently they also tested whether there is a functional interaction between native TRPP2 and TRPC1 in murine IMCD cells, where cells were either transiently transfected with TRPC1 alone or TRPC1 co-transfected with TRPP2 (gain-of-function), or transfected with TRPP2-D511V mutant, I-mfa, a known inhibitor of TRPC1-specific current, or TRPC1-specific shRNA construct (loss-of-function). The results obtained from perforated patch-clamp in these cells suggest that native functional TRPP2/TRPC1 channel complex may exist in the cells [3]. Furthermore, using TRPP2 and TRPC1 reconstituted in lipid bilayer, Zhang and co-authors have revealed that TRPP2 and TRPC1 functional interact to form a heteromultimer with the biophysical properties of the channel similar to what Bai et al. described [4]. It has also been reported that TRPP2 interacts with TRPC1 to form a heterotetramer with a 2:2 stoichiometry [5]. However, nearly all of the evidence for a functional association between TRPP2 and TRPC1 has been based upon transfection/over-expression of these TRP channel components or upon reconstitution of these channels into lipid bilayer [3–5].

Recent studies have also reported that TRPP2 and TRPV4 colocalize to the cilia of polarized madin-darby canine kidney epithelial (MDCK) cells [6]. They concluded that TRPP2 utilizes TRPV4 to form a mechano- and thermosensitive molecular sensor in the cilium, based upon the results from studies of flow-induced Ca^2+^ transients in MDCK cells and a tail immersion assay in TRPP2^+/-^ mice and TRPV4^-/-^ mice. Subsequently these investigators tested whether TRPP2 and TRPV4 interact functionally, where whole-cell current was measured in *Xenopus* oocytes expressing these channels. Coexpression of TRPP2 and TRPV4 significantly amplified the swelling-activated and warm temperature-activated currents; whereas steady-state currents were not altered suggesting TRPP2 alters the functional properties of TRPV4. Similar results were observed in HEK293 cells coexpressing these channels [6]. Interestingly, using atomic force microscopy Stewart et al. revealed that TRPP2 and TRPV4 assemble as a heterotetramer with a 2:2 stoichiometry; however, there was a lack of functional evidence regarding whether these two proteins function as an ion channel [7]. Thus although there is some indication that TRPP2 and TRPV4 may form a channel complex in renal epithelial cells there is a lack of clear biophysical evidence for this channel, a paucity of information on channel regulation and whether this channel exists in collecting duct cells which are a common site for cystic development in PKD.

In the present studies we identified an endogenous non-selective divalent cation-permeable channel with a single-channel conductance of ~23-pS. This channel equally conducts Ba^2+^ in either direction across the plasma membrane. Interestingly, the rate of occurrence of this channel was much higher in cilia cells (-) compared to cilia (+) cells. The ability of this channel to conduct divalent cation and the higher occurrence rate in cilia (-) cells may result in chronic elevations of [Ca^2+^]_i_ in cilia (-) cells as has been previously reported [13]. Although we detected mRNAs of TRPP1, TRPP2, TRPV4, TRPM4, TRPM6, TRPC1 and TRPC2 in both cell types, however, this 23-pS channel exhibits distinct biophysical features compared to the individual TRP channels detected by RT-PCR, as well as to TRPV5 and TRPV6 [3–5,21–23,28–31]. We hypothesized that this channel might be a heterotetramer of TRPP2 and TRPV4. Co-IP data demonstrated that these two proteins physically interact in both cell types. To determine if this finding is unique to this particular cell line we also assessed protein interactions in IMCD cells, which is a commonly used renal epithelial cell line. We found this same association between TRPP2 and TRPV4 in this cell line (Figure **S3b**), strengthening the generality of our findings. Furthermore, these two proteins are not only co-localized to the apical membrane but also at the cilium, which is consistent with the results obtained by Köttgen and co-workers, where native TRPP2 and TRPV4 are co-localized at the cilium in polarized MDCK cells [6]. Our functional studies showed that when TRPP2 was knocked down there was the occurrence of the channels with the biophysical properties very similar to TRPV4 channels [25,26] and with TRPV4 knocked down there was the appearance of a channel having the biophysical properties of TRPP2 [25]. We have previously reported the occurrence of a ~80-pS channel in this cell line, however, the frequency of detection of this channel was exceedingly rare, which is quite consistent with the results obtained in this study (Table **1**) [13]. In this previous study we focused on channels that were within the ~80-pS range since this is the reported size of the TRPP2 channel. It is quite conceivable that not all of TRPP2 channels are complexed with TRPV4 and that knockdown TRPV4 results in a greater appearance rate of ~80-pS channel in the patches (Table **1**; Figure **5h**), which could explain our previous findings. Moreover, compared to wild-type cells in either TRPP2 or TRPV4 knockdown cells, the appearance rate of the 23-pS channel was greatly reduced from ~60% to ~10% and 18%, respectively; it was further decreased in TRPP2\TRPV4 double knockdown cells to ~5%. Taken together, these data strongly support the notion that endogenous TRPP2 and TRPV4 assemble to form a ~23-pS, calcium permeable non-selective cation channel.

### EGF stimulates TRPP2\TRPV4 channel complex via ligand-receptor interaction

Consistent with the notion that there is mislocalized EGFR and increased activity of EGFR in PKD [9], we found that EGFR was mislocalized to the apical membrane in cultured cilia (-) cells (data not shown). In addition, there appeared to be a greater abundance of EGFR protein and p-ERK in cilia (-) cells compared to cilia (+) cells in the absence of EGF. With EGF-stimulation there was a time-dependent decay of EGFR expression level perhaps through internalization and degradation, which is consistent with what is known for ligand-EGFR interactions [19,20]. In contrast, EGFR remained at a constant level in cilia (-) cells with apical EGF for at least for 3 hrs. Ligand-induced EGFR internalization does not occur at a normal rate in cilia (-) cells, which could be explained by the fact that subunits of EGFR are expressed at the apical membrane [10]. Since cystic fluid is filled with biologically active ligands for EGFR [9], this delayed internalization of EGFR in cilia (-) cells would lead to inappropriate sustained EGF signaling.

Previous work has reported that TRPP2 was up-regulated by EGF in LLC-PK cells transfected with TRPP2 and that TRPP2 and EGFR co-localized in the cilia of LLC-PK cells [11]. We demonstrate that EGF application also markedly up-regulated the 23-pS TRPP2\TRPV4 channel. This channel activity was significantly increased by basolateral administration of EGF in cilia (+) cells and by apical application of EGF in cilia (-) cells. However, the up-regulation of TRPP2\TRPV4 channels in cilia (-) cells by EGF was almost 4-fold larger than that in cilia (+) cells. Furthermore, EGF-induced activation of TRPP2/TRPV4 channel and the abundance of p-ERK were greatly attenuated by either a tyrosine kinase inhibitor or a MAP kinase inhibitor, which suggest that the downstream signaling of ligand-EGFR interactions account for the aberrant activity of TRPP2/TRPV4 channel at the apical membrane. Therefore, we speculate that there may be a high level of Ca^2+^ channel activity at the apical membrane of cilia (-) cells that occurs, in part, due to sustained apical EGFR activation. This finding was further supported by enhanced divalent cation entry in cilia (-) cells as assessed by the Mn^2+^-quenching assay [32].

### TRPP2\TRPV4 mediated Ca^2+^ influx contributes to hyperproliferation in cilia (-) cells

We tested whether the EGF could directly enhance divalent cation entry using a well-established method; the Mn^2+^-quenching assay [32]. Our data suggest that EGF promotes Ca^2+^ influx in cilia (-) cells but not in cilia (+) cells. Furthermore, knock down either TRPP2 or TRPV4 or double knock down of TRPP2\TRPV4 appeared to reduce EGF-induced Ca^2+^ influx in cilia (-) cells. In addition, cell proliferation rate was significantly higher in cilia (-) cells under control conditions; when cells were incubated with apical EGF the rate of cell proliferation were significantly up-regulated in cilia (-) cells but not in cilia (+) cells. This is, most likely, due to the fact that the basolateral membrane EGFR was not accessible to the ligand. The rate of cell proliferation in cilia (-) cells was greatly attenuated by BAPTA/AM, an intracellular Ca^2+^ chelator, and EGFR-dependent signaling inhibitors, suggesting that TRPP2\TRPV4 mediated increase in [Ca^2+^]_i_ may play a key role in EGF-induced hyperproliferation in cilia (-) cells. This is supported by the finding that EGF-induced increase in cell proliferation was significantly inhibited by knock down of both TRPP2 and TRPV4 proteins.

Previous studies have suggested that cyst formation is associated with reduced [Ca^2+^]_i_ [33,34]. However, it has recently been reported that overexpression of TRPP2 can also lead to cyst formation [35,36]. In addition, TRPV4-deficient animal models do not develop cysts suggesting that TRPV4, although an essential component of the ciliary mechanosensor *in vivo*, its absence is not sufficient for cystic development [6]. Thus, the relationship between TRPP2, TRPV4, Ca^2+^, and cystogenesis is complex. Our results are consistent with the view that during the development of cystogenesis, the presence of EGFR ligands would result in the activation of mislocalized apical EGF receptors, which would enhance the Ca^2+^ influx via aberrant activity of TRPP2\TRPV4 channels and would subsequently lead to augmented cell proliferation and cystic formation. 

## Supporting Information

Text S1 (DOC)

Table S1 These primers were used for RT-PCR to detect mRNAs of targeting genes as listed in the Table 1.
(DOC)

Table S2 **Information of TRPP2- and TRPV4-specific shRNAs.** The constructs were purchased from OPEN Biosystems.
(DOC)

Figure S1 (TIF)

Figure S2 (TIF)

Figure S3 (TIF)
