# Supplementary material for: TRPP2 and TRPV4 Form an EGF-Activated Calcium Permeable Channel at the Apical Membrane of Renal Collecting Duct Cells
Source: PLoS One. 2013 Aug 16;8(8):e73424. doi: 10.1371/journal.pone.0073424 (PMC3745395; doi:10.1371/journal.pone.0073424)
Supplement: Text S1 — (DOC) [file pone.0073424.s001.doc]

**SUPPLEMENT RESULTS**

**Recording of TRPP2\TRPV4 channel using excised inside-out patch configuration**

**Fig S1** demonstrates the single-channel traces that were recorded from a cilia (-) cells at the membrane potentials (V_M_) ranged between 100 mV to-100 mV.

**Pharmacological profiles of TRPP2\TRPV4 channels.**

To rule out the possibility that the currents recorded are mediated by TRPM4 or ENaC, 2 mM ATP or 10µM amiloride was applied to the patches. Activity of TRPP2\TRPV4 channel was not inhibited by additional 2 mM ATP (**Fig S2a**). In addition, 10 µM amiloride did affect the activation of TRPP2\TRPV4 channels (**Fig S2b**). These results along with the ability of the channels to conduct Ca^2+^ suggest that this channel was not mediated by TRPM4 channels or ENaC.

**RT-PCR detect the presence of TRP genes in cilia (+) and cilia (-) cells.**

Among the tested genes (**Table S1**) TRPP1, TRPP2，TRPV4 TRPM4，TRPM6，TRPC1 and TRPC2 were detected by reverse transcript PCR analysis (**Fig S3a & S3b**) in both cell types. However, the rest tested members of TRP family were not detected (dada not shown).

**TRPP2 and TRPV4 interact in IMCD cells.**

Co-IP assays demonstrated that TRPP2 associates with TRPV4 in polarized inner medullary collecting duct (IMCD) cells (**Fig S3c**). This result confirms that association of TRPP2 and TRPV4 is not unique to cilia (+) and cilia (-) cells

**SUPPLEMENT** **MATERIALS AND METHODS**

**Electrophysiology.**

Refer to Material and Methods section in the main article.

**Co-IP in IMCD cells.**

IMCD cells were lysed with Cell Lytic M Cell Lysis Reagent (Sigma) with 1x protease inhibitor cocktail EDTA-free (Roche). Cell lysates were pre-cleared with immobilized Protein G (PIERCE) for 30 min at 4^0^C. Supernatants were incubated with 1µg of anti-TRPV4 (Santa Cruz #47527) antibody or anti-TRPP2 antibody with gentle agitation at 4^0^C overnight. The next day Immobilized Protein G was added to the lysates followed by gentle rotation at 4^0^C for two hours. After centrifugation at 4^0^C, beads were washed four times with washing buffer (50% Cell Lytic M Cell Lysis Reagent/50% PBS + protease inhibitors) and finally dissolved in 2X SDS sample buffer. Samples were then prepared for SDS-PAGE and Western blot analysis by boiling for five min and spun briefly to pellet the beads and analysis was performed on the supernatant.
