# Supplementary material for: TRPP2 and TRPV4 Form an EGF-Activated Calcium Permeable Channel at the Apical Membrane of Renal Collecting Duct Cells
Source: PLoS One. 2013 Aug 16;8(8):e73424. doi: 10.1371/journal.pone.0073424 (PMC3745395; doi:10.1371/journal.pone.0073424)
Supplement: Table S1 — These primers were used for RT-PCR to detect mRNAs of targeting genes as listed in the Table 1. (DOC) [file pone.0073424.s002.doc]

**Supplement Table1**

Primer F1(5^，^- 3^，^) R1(5^，^- 3^，^)

Name

TRPV4 ACA ACA CCC GAG AGA ACA CC CCC AAA CTT ACG CCA CTT GT

TRPV5 TGT CAT CCT AGA CTG GCT CCT GAG CAG CCT CCA TCA GCA TT

TRPV6 ACA CAA GCC CAG CAG ATT TC CAT TAG CAC CAT TGC AGC CTC

TRPP1 CTT GGT GTG GCC TAT GCA CA TGA AGC TTC TGA GCC TGA GC

TRPP2 TCC CCA GAA GCC TGG ATG AC TTT GCG AAG CTG CAT CAT CC

TRPM4 TGG ATG CTC TGC TGA ATG AC GAC TCT AGG CGA GCC ATC AC

TRPM6 GCT GAT TGA AGA ACA CCA TG CCA TAC CCA GAG GAT GAC

TRPC1 ATT CCA CCC CAC GCT TGT AG GCA AAG CAG GTG CCA ATG AA

TRPC2 ATG TTC GGC ATG GAA GAG CA GAT GAC TCG AAG GCG GTA GG

TRPC3 AGA GCG ATC TGA GCG AAG TC TTT GGA ACG AGC AAA CTT CC

TRPC4 ACG CGT TTT CCA CGT TAT TC CTT CGG TTT TTG CCT CTC TG

TRPC5 GGT AGA TAG TCT ACG TCA TTC CAC CAA TCA TGG ATG TAT TC

TRPC6 GCT CAT CCA AAC TGT CAG CA CAG CAT TCC AAA GTC AAG CA

TRPC7 AGG GAT AAG TGG TGG CCT T TGA ACT TGG AGT TCA GCA TG

These primers were used for RT-PCR to detect mRNAs of targeting genes as listed in the Table 1.
