# Supplementary material for: TRPP2 and TRPV4 Form an EGF-Activated Calcium Permeable Channel at the Apical Membrane of Renal Collecting Duct Cells
Source: PLoS One. 2013 Aug 16;8(8):e73424. doi: 10.1371/journal.pone.0073424 (PMC3745395; doi:10.1371/journal.pone.0073424)
Supplement: Table S2 — Information of TRPP2- and TRPV4-specific shRNAs. The constructs were purchased from OPEN Biosystems. (DOC) [file pone.0073424.s003.doc]

**Supplement Table 2**

| **Plasmid Name** | **Selection** | **Location** | **Description/Notes** | **Sequences** |
| --- | --- | --- | --- | --- |
| TRPV4 1  (shRNA1) | Amp | A-1 | TRCN0000068618 | 5' AAACCCAGGGCTGCCTTGGAAAAG 3' |
| TRPV4 2  (shRNA2) | Amp | A-1 | TRCN0000068619 | 5' AAACCCAGGGCTGCCTTGGAAAAG 3' |
| TRPV4 3  (shRNA3) | Amp | A-1 | TRCN0000068620 | 5' AAACCCAGGGCTGCCTTGGAAAAG 3' |
| TRPP2 1  (shRNA1) | Kan/Cm | A-1 | V2MM_45609 | 5' TGT GGA AAG GAC GAA ACA CC 3' |
| TRPP2 2  (shRNA2) | Kan/Cm | A-1 | V2MM_49580 | 5' TGT GGA AAG GAC GAA ACA CC 3' |
| TRPP2 3  (shRNA3) | Kan/Cm | A-1 | V2MM_52971 | 5' TGT GGA AAG GAC GAA ACA CC 3' |
| pLKO.1 empty vector control | Amp | A-1 | pkd1/TRPV4 negative control | No sequences |

Information of TRPP2- and TRPV4-specific shRNAs. The constructs were purchased from OPEN Biosystems.
