# Supplementary figures and images for: TRPP2 and TRPV4 Form an EGF-Activated Calcium Permeable Channel at the Apical Membrane of Renal Collecting Duct Cells
Source: PLoS One. 2013 Aug 16;8(8):e73424. doi: 10.1371/journal.pone.0073424 (PMC3745395; doi:10.1371/journal.pone.0073424)

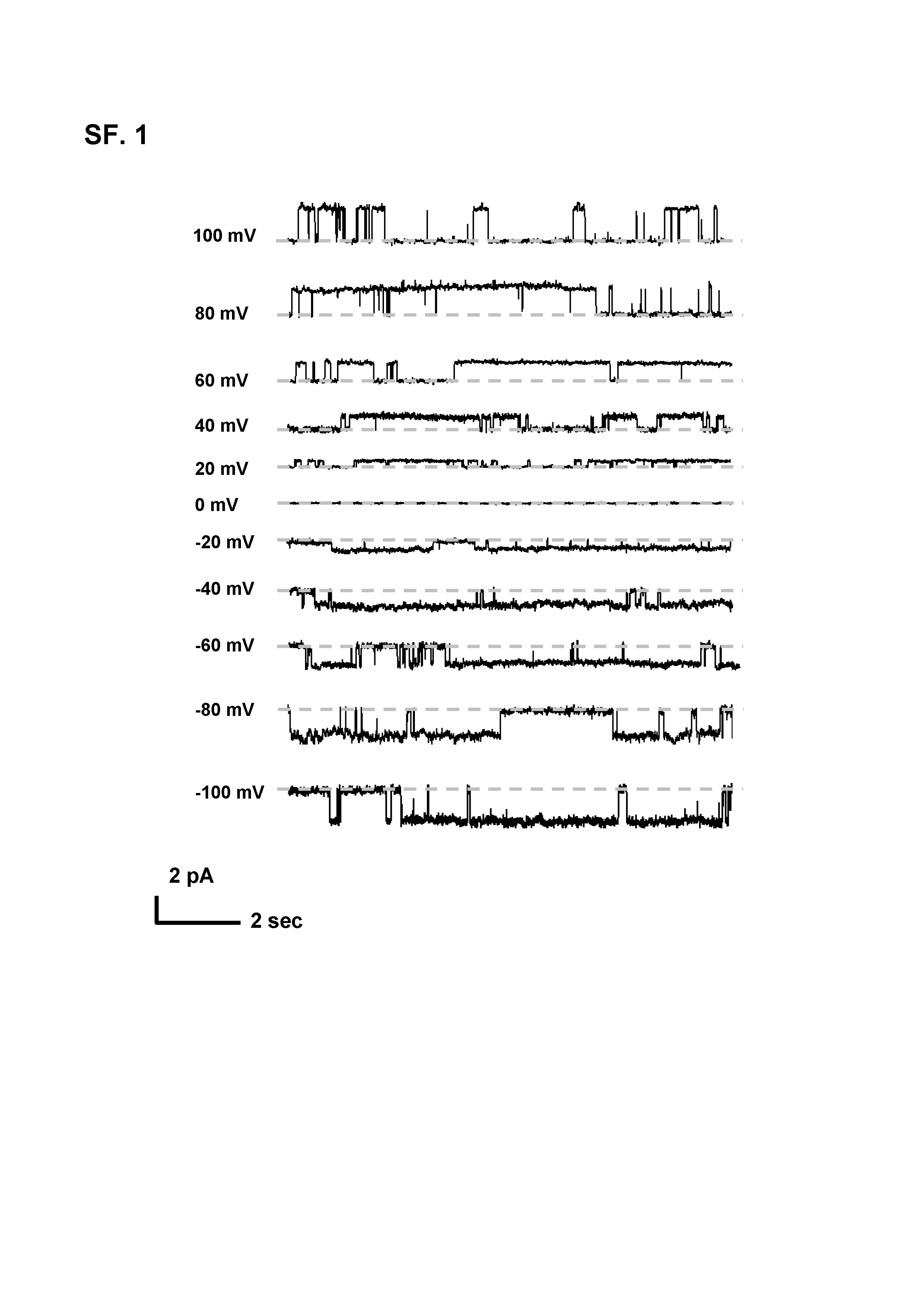

Supplement: Figure S1 — (TIF) [file pone.0073424.s004.tif]

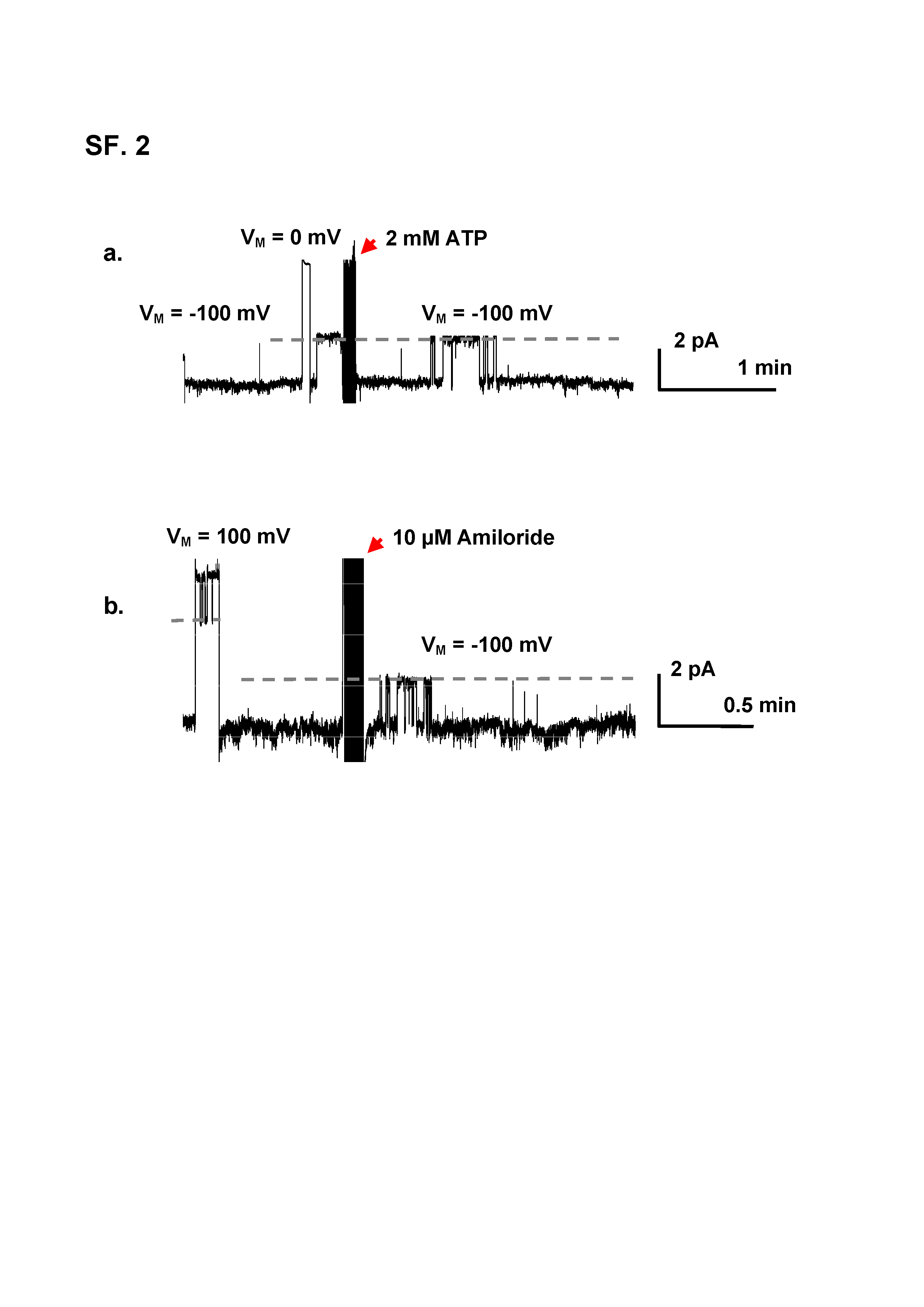

Supplement: Figure S2 — (TIF) [file pone.0073424.s005.tif]
